# Editorial: Protein–Protein Interactions: Drug Discovery for the Future

**DOI:** 10.3389/fchem.2021.811190

**Published:** 2021-11-29

**Authors:** Simona Rapposelli, Eugenio Gaudio, Fabio Bertozzi, Sheraz Gul

**Affiliations:** ^1^ University of Pisa, Pisa, Italy; ^2^ DTI-Tech, Bellinzona, Switzerland; ^3^ Istituto Italiano di Tecnologia (IIT), Genova, Italy; ^4^ Fraunhofer Institute for Translational Medicine and Pharmacology, Hamburg, Germany

**Keywords:** drug discovery, pharmacology, proteins, assays, protein-protein interaction (PPI)

The human proteome is comprised of approximately 20,000 proteins and significantly more protein-protein interactions (PPIs) that play pivotal roles in biological processes. Their dysregulation often results in the onset and progression of several diseases. PPIs therefore represent a treasure trove of disease modifying drug targets-However, targeting these is a challenging task when attempting to convert drug-like small molecules to therapeutics. When targeting PPIs, it is necessary to have a balance between the interacting proteins to provoke a therapeutic and not a significant adverse effect. This is elegantly illustrated by degradation of TP53 mediated by MDM2 which is prevented by Nutlin-3, in addition to other relevant PPIs which have been discovered (Gul and Hadian, 2014).

A number of high quality articles relating to PPIs are reported in this Protein-Protein Interactions: Drug Discovery for the Future Research Topic that make use of a variety of experimental techniques. As mentioned above, the number of PPIs are vast, the methods proposed by Lawson et al. and Martino et al., allow the mapping and elucidating those PPIs that can be targeting by small molecules. This is particularly relevant in order to identify those PPIs which should be the focus of drug discovery efforts. For example, the TCL1 (T-Cell Leukemia/Lymphoma 1) oncogene and FHIT (Fragile Histidine Triad Diadenosine Triphosphatase) are relevant drug targets in cancer and their interacting partners have been studied extensively (Gaudio et al., 2012; Gaudio et al., 2013a; Gaudio et al., 2013b; Paduano et al., 2018). In many cases the interacting partners in PPIs are poorly understood and González-Avendaño et al. used proteomics approaches based on mass spectrometry to resolve complex interacting partners of well characterized proteins. Each novel PPI was further investigated with the aim of understanding its importance in the biology and biochemistry of cancer. It was also shown as PPIs are functional to signaling pathways that are up-regulated in cancer, such as PI3K-AKT, NFκB and drug resistance.

Liang et al. used a drug discovery approach to consider not only drug activity and selectivity, but also drug-like properties and the associated primarily toxicity. To this end, the synthesis of new drug-like small molecules or the design of close analogues, starting from natural products was undertaken in order to allow for suitable compound optimization. Chemical modifications, starting from the planar marine natural product fascaplysin, led to the identification of nonplanar tetrahydro-β-carboline analogs with conserved capability to bind selectively its target CDK4. The synthetically modified derivatives of the natural product resulted reduced toxicity using *in vitro* models.

Parate et al. used a combination of the computational methods, biological and biophysical validations allow the realization of compelling studies of both drug-target and protein-protein interactions. By applying computational chemistry and Molecular Dynamics Simulations, the most relevant hot-spot between the Raf kinase inhibitory protein (RKIP) and C-Raf (Raf-1 kinase) was identified as a potential target of therapy.

Wang et al. report on the generation of peptides by a computational proposed synthesis or selected by phage display. The specific activity towards PPIs proposes peptides as chemical scaffolds amenable of further modifications in order to originate peptidomimetics with drug-like small molecule characteristics. This approach allowed the selection of a short FHIT peptide who recapitulated the function of the full length protein in sequestering annexinA4 in the cytosol and prevents its activity as pump of drug resistance (Gaudio et al., 2016). By performing such research to study their Mechanism of Action (MoA), we will have the chance to see compounds working through a novel mechanism, find new targets and even more new PPIs responsible for the disease. This will be accelerated by using genetics and proteomics approaches together with biochemical and biophysical methods to validate the mentioned interactions.

In order to understand the role of PPIs in disease, the appreciation of a variety of technologies is required. However, serendipity is still an important player in drug discovery and this is illustrated by the KRAS inhibitor recently discovered by Amgen which is now opening a new era in what was until recently considered an undruggable drug target. This is as a consequence of the progress in computational chemistry and in the evaluation of old and new compounds with drug-like potential. Although structure-based design has played a key role in the drug discovery process, with three dimensional structures guiding medicinal chemistry efforts and the apt metaphor of *a lifting bridge over a canal,* the spatial-temporal relationship highlighted by protein dynamics may help to increase the probability of discovering small molecules that can stabilize interesting conformers of target proteins.

The successful design of PPI modulators is still a great challenge, and is generally supported by a detailed knowledge of the system at molecular and structural level. Structural and biophysical methods for ligand discovery targeting PPIs, supported by the identification and validation of protein-protein complexes, are valuable tools to possibly tackle difficult PPIs. With this, X-ray crystallography and cryoEM are essential to successfully assess the structural and mechanistic details of interaction events at atomic resolution, and key for the development of novel PPI modulators.

With the resurgence of phenotypic assays in drug discovery, the observations in these systems are often complex as recently shown by cell-cell interactions in stroke models (Kikuchi-Taura et al., 2020; Takeuchi et al., 2020; Kikuchi-Taura et al., 2021) and understanding the nature of the PPIs using novel technologies will allow the scientific community to better understand and unveil therapeutically relevant aspects of such PPIs to treat a variety of human diseases.

## References

[B1] Gaudio E. Spizzo R. Paduano F. Luo Z. Efanov A. Palamarchuk A. (2012). Tcl1 Interacts with Atm and Enhances NF-κB Activation in Hematologic Malignancies. Blood 119, 180–187. 10.1182/blood-2011-08-374561 22065599 PMC3251228

[B2] Gaudio E. Paduano F. Ngankeu A. Lovat F. Fabbri M. Sun H.-L. (2013a). Heat Shock Protein 70 Regulates Tcl1 Expression in Leukemia and Lymphomas. Blood 121, 351–359. 10.1182/blood-2012-09-457374 23160471 PMC3544116

[B3] Gaudio E. Paduano F. Spizzo R. Ngankeu A. Zanesi N. Gaspari M. (2013b). Fhit Delocalizes Annexin A4 from Plasma Membrane to Cytosol and Sensitizes Lung Cancer Cells to Paclitaxel. PLoS One 8, e78610. 10.1371/journal.pone.0078610 24223161 PMC3819369

[B4] Gaudio E. Paduano F. Ngankeu A. Ortuso F. Lovat F. Pinton S. (2016). A Fhit-Mimetic Peptide Suppresses Annexin A4-Mediated Chemoresistance to Paclitaxel in Lung Cancer Cells. Oncotarget 7, 29927–29936. 10.18632/oncotarget.9179 27166255 PMC5058653

[B5] Gul S. Hadian K. (2014). Protein-protein Interaction Modulator Drug Discovery: Past Efforts and Future Opportunities Using a Rich Source of Low- and High-Throughput Screening Assays. Expert Opin. Drug Discov. 9, 1393–1404. 10.1517/17460441.2014.954544 25374163

[B6] Kikuchi-Taura A. Okinaka Y. Takeuchi Y. Ogawa Y. Maeda M. Kataoka Y. (2020). Bone Marrow Mononuclear Cells Activate Angiogenesis via Gap Junction-Mediated Cell-Cell Interaction. Stroke 51, 1279–1289. 10.1161/STROKEAHA.119.028072 32075549

[B7] Kikuchi-Taura A. Okinaka Y. Saino O. Takeuchi Y. Ogawa Y. Kimura T. (2021). Gap junction-mediated Cell-Cell Interaction between Transplanted Mesenchymal Stem Cells and Vascular Endothelium in Stroke. Stem Cells 39, 904–912. 10.1002/stem.3360 33704866 PMC8807299

[B8] Paduano F. Gaudio E. Mensah A. A. Pinton S. Bertoni F. Trapasso F. (2018). T-Cell Leukemia/Lymphoma 1 (TCL1): An Oncogene Regulating Multiple Signaling Pathways. Front. Oncol. 8, 317. 10.3389/fonc.2018.00317 30151355 PMC6099186

[B9] Takeuchi Y. Okinaka Y. Ogawa Y. Kikuchi-Taura A. Kataoka Y. Gul S. (2020). Intravenous Bone Marrow Mononuclear Cells Transplantation in Aged Mice Increases Transcription of Glucose Transporter 1 and Na+/K+-ATPase at Hippocampus Followed by Restored Neurological Functions. Front. Aging Neurosci. 12, 170. 10.3389/fnagi.2020.00170 32595487 PMC7301702

